# Perceived a community with shared future for doctor-patient and benefit finding: a moderated mediation model 

**DOI:** 10.1186/s40359-023-01175-6

**Published:** 2023-05-30

**Authors:** Renjie Lu, Shenyu Zhao, Jing Zhou, Weiyan Ou, Juan Wen, Lingmin Hu

**Affiliations:** 1 Changzhou Third People’s Hospital, Changzhou Medical Center, Nanjing Medical University, Jiangsu, Changzhou, 213000 China; 2 Business School, ISCTE University Institute of Lisbon, Lisbon, 1649-026 Portugal; 3 School of Health Management, Southern Medical University, Guangdong, Guangzhou, 510515 China; 4 Department of Neurology, Changzhou Third People’s Hospital, Changzhou Medical Center, Nanjing Medical University, Jiangsu, Changzhou, 213000 China; 5 Department of Reproduction, Changzhou Maternity and Child Health Care Hospital, Changzhou Medical Center, Nanjing Medical University, Jiangsu, Changzhou, 213000 China; 6 Nanjing Maternity and Child Health Care Institute, Women’s Hospital of Nanjing Medical University, Nanjing Maternity and Child Health Care Hospital, Jiangsu, Nanjing, 210000 China

**Keywords:** Perceived a community with shared future for doctor-patient, Benefit finding, Anxiety, Health self-consciousness, Moderating effect, Mediating effect

## Abstract

**Background:**

Under the background that the concept of a community with shared future for mankind has been advocated, the doctor-patient relationship has rapidly sublimated into a community with shared future for doctor-patient. The purpose of this study was to analyze the changes and relationships of anxiety, perceived a community with shared future for doctor-patient (PCSF), health self-consciousness (HSC) and benefit finding (BF) in the outbreak stage of COVID-19 and in the stable stage of COVID-19.

**Methods:**

The questionnaire consisted of a self-designed health self-consciousness scale, perceived a community with shared future for doctor-patient scale, revised 7-item generalized anxiety disorder scale and benefit finding scale. Questionnaires were administered in the outbreak stage of COVID-19 and in the stable stage of COVID-19 to address public anxiety, BF, and trust between medical staff and patients.

**Results:**

Risk perception will increase anxiety in public, and the public who trust medical staff and the ability of the government to prevent and control the epidemic will have a higher PCSF. Compared with those in the outbreak stage of COVID-19, PCSF, HSC and BF all decreased in the stable stage of COVID-19. HSC partly plays a mediating role in the process of the influence of PCSF and BF (95% CI = [0.3785, 0.5007], [0.2357, 0.3695], *P* < .001). The R-value of the model in the outbreak stage of COVID-19 and in the stable stage of COVID-19 were 0.555 and 0.429, and the value of R^2^ was 0.308 and 0.184 respectively (*P* < .001). In the stable stage of COVID-19, the coefficient of anxiety ✕ PCSF is negative. The B values of anxiety and PCSF are positive, and the moderating effect is negative (*P* = .038). Anxiety has a negative moderating effect between PCSF and HSC, indicating that anxiety will weaken the positive impact of PCSF on HSC. It means that there exists a substitution relationship between anxiety and PCSF.

**Conclusions:**

The common goal of medical staff and patients is health, and health is the premise of the meaning of life. Vigorously advocating for PCSF can not only promote a harmonious doctor-patient relationship, but also establish a good HSC and improve the understanding of the meaning of life in the public. Furthermore, if the common concept of a community with a shared future for doctor-patient is integrated into the values of life, it may be more stable and long-term to maintain a good doctor-patient relationship. In addition, we should guard against the influence of high-level anxiety on the path of meaning perception.

**Supplementary Information:**

The online version contains supplementary material available at 10.1186/s40359-023-01175-6.

## Background

Corona Virus Disease 2019 (COVID-19) is the common enemy of all mankind [1]. The outbreak of COVID-19 highlights the difficulties the global faces in dealing with public health problems, such as the lack of collaboration among governance bodies [1]. To better respond to the epidemic, the concept of a community with shared future for mankind has been mentioned, that is, all partners and national leaders of the international community to take strategic action as soon as possible to fight COVID-19 together [2]. These practices of solidarity and mutual assistance apply not only between different countries but also between different communities and groups, such as medical staff and patients. Generally speaking, the community with shared future for doctor-patient is that both medical staff and patients have the same understanding of the disease, make efforts to deal with the disease in the same direction, and share the responsibility for the results of treating disease. Perceived a community with shared future for doctor-patient (PCSF) means that the perceived interests of both medical staff and patients are generated in the process of mutual understanding, joint efforts to cope with diseases and assuming responsibilities. Under the influence of the common goal of fighting the epidemic, the two groups of medical staff and patients have shifted from opposing interests to responding to COVID-19 in unison. In the outbreak stage of COVID-19, Chinese medical staff have shown resilience and professional dedication in overcoming difficulties [3]. The doctor-patient relationship in China has been improved [4]. The public has confidence in the long-term development of the doctor-patient relationship [5].

COVID-19 is transmitted mainly through droplets, respiratory secretions and direct contact [6]. This way of transmission has also led to the rapid outbreak of the epidemic around the world, causing serious public panic. The risk perception described that risks are perceived as more dangerous when they are uncommon [7–11]. The low predictability of the COVID-19 course could increase risk perception [12]. Studying the changes in perceived risk of self-infection during the development of the epidemic, it can reflect the changes in public psychological panic. Related research shows that perceived risk of self-infection and worry about self-infection are positively correlated with the level of anxiety [13]. The epidemic has made the public take more heed of personal health problems and the public start to pay attention to the relevant health care knowledge and concepts to maintain their health, which shows a clear increase in public health awareness, and public health consciousness has been significantly increased [14]. And this health self-consciousness (HSC) may be affected by the anxiety generated by risk perception. And self-consciousness refers to the extent to which people direct their attention inward or outward and it is an important behavioral determinant of social interaction [15]. When perception exceeds the threshold of psychological tolerance, the status of its influence on HSC gradually increases and ensuring one’s own health becomes the most important goal, while the status of the influence of other factors on HSC may decrease. When individuals change their HSC, they will perform one healthy behavior, which may promote other healthy behaviors. Some studies have confirmed that there is a consistency between health consciousness and health behavior [16]. We support the view that this cross-behavioral associations may be due to the existence of intergoal facilitation [17] and common underlying motivation between behaviors [18]. The reason for the cross-behavioral associations is the intercommunication of different consciousnesses. We speculate that the good health consciousness established by the epidemic, as a positive way of thinking, may have a positive effect on individual life values through the “common mechanism”.

Health problems are the biggest negative threat to individuals. At the beginning of the epidemic, the threat posed by COVID-19 to the public was unknown. Fear and uncertainty can make people feel stressed, anxious and debilitated [19]. About one-third of the respondents reported moderate to severe anxiety [20]. As everything has two sides, we believe that after two years of the epidemic, it will not only cause anxiety and depression among individuals, but also provide opportunities for positive changes in the spirit and interpersonal relationships of individuals [21]. Health is not only the goal that life devotes to pursue, but also the intrinsic value of life. Negative experiences can better enhance the meaning of life by stimulating comprehension (understanding how the event fits into a broader narrative of the ego, relationships and the world) among people [22]. Perceived solidarity among interdependent social groups may make life meaningful [23]. For example, medical staff and patients unite in response to the epidemic. Prosocial motivation is related to compliance with healthy behavior and the habit of keeping social distance [24]. PCSF in public is not only a higher level of understanding of the doctor-patient relationship, but also a prosocial motivation.

A study divided COVID-19 in China into four stages, which was the incubation stage, the outbreak stage, the resolution stage and the stable stage. Among them, the outbreak stage refers to the daily increase in the number of new infections and the spread of the epidemic throughout the country, and the stable stage refers to the effective control of the epidemic through various measures [25]. Similarly, when a city is dealing with an epidemic, the development of the epidemic will also go through the process from the outbreak stage to the stable stage. The two surveys of this study were carried out at the stage of China’s regular prevention and control of COVID-19. The first survey was conducted at the outbreak stage of COVID-19 in the city and this stage was described as “the outbreak stage of COVID-19” in our study. The second survey was carried out immediately after there were no new infected persons in the city for 21 consecutive days (3 weeks), which was described as “the stable stage of COVID-19”.

Anxiety, PCSF, HSC and benefit finding (BF) are four variables related to the epidemic. The relationship between them will change with the occurrence and development of the epidemic. In addition, they will bring people psychological stress and spiritual benefits. The psychological stress response may last for a short time, but the mental benefit may remain stable for a long time. Exploring the relationship between them is helpful to understand people’s psychological state under the background of epidemic situation and guiding people to carry out effective psychological intervention. Furthermore, shaping people’s values through the common concept of doctor-patient fate can promote harmonious doctor-patient relationship. Although these four variables closely related to the occurrence and development of the epidemic, there is no literature review in the relevant field that describes the relationship between these four variables in the context of the epidemic. There is a literature involved risk perception and the doctor-patient relationship, but there is no clear indication of the relationship between them [26]. This is the innovation of this research. So, we chose the two periods of time to conduct a longitudinal study to analyze the relationship among anxiety, PCSF, HSC and BF in China, which were the outbreak stage of COVID-19 and stable stage of COVID-19. We make the following assumptions:

H1: Anxiety is affected by perceived risk of the epidemic.

H2: PCSF is affected by the trust of the public in medical staff and patients and the ability of the government in epidemic prevention and control.

H3: Compared with those in the outbreak stage of COVID-19, the anxiety, PCSF, HSC and BF decrease in the stable stage of COVID-19.

H4: There is correlation among anxiety, PCSF, HSC and BF.

H5: PCSF will positively affect the BF, and HSC and plays a mediating role.

H6: PCSF has a positive impact on HSC, and anxiety plays a moderating role.

## Methods

### Study design and setting

This was a longitudinal observational study. We produced an electronic questionnaire and distributed it in designated cities on the Internet through the “Questionnaire Star” platform (https://www.wjx.cn). The survey cities are distributed in all directions in China and are geographically representative. The first survey was conducted from November 13, 2021 to November 20, 2021 in cities, which included Beijing, Dalian, Zhengzhou, Heihe and Shangrao, which were experiencing the epidemic during the investigation period. The second survey was conducted from December 1 to December 19 of the same year in cities, which included Dalian, Zhengzhou, Heihe, Shangrao and Lanzhou. COVID-19 occurred in all surveyed cities during the first period. The second survey was conducted on the first day after three consecutive weeks of no new infections in the city (the 22nd day since the city has no new infections). The informed consent of the respondents should be obtained before filling out the questionnaire. All respondents who were 18 years old or older and not medical staff were eligible to participate in this survey. Those who object to the terms of informed consent and not have the ability to think independently will be suspended and excluded from the survey. In addition, the questionnaires filled by the respondents with low quality will be excluded through the quality control of questionnaires.

### Study sample size estimation

The sample size was calculated through the G*power 3.1.9.7 software with a statistical power of 80%, and a significance level of 0.05 [27]. Employing the two-tailed t-tests, the required sample size was 105 respondents in each group at least. The total number of respondents required for both groups was at least 210 respondents.

### Data collection and procedure

To reduce the risk of exposure to the virus, we produced an electronic questionnaire online and distributed it in two time periods through a convenient sampling survey. Then we conducted quality control on the collected questionnaires to judge their validity. In the first survey conducted in cities including Beijing, Dalian, Zhengzhou, Heihe and Shangrao, a total of 1534 questionnaires were collected from November 13, 2021 to November 20, 2021 and 1252 were valid, with an effective rate of 81.62%. In the second survey conducted in cities including Dalian, Zhengzhou, Heihe, Shangrao and Lanzhou, a total of 1075 questionnaires were collected from December 1, 2021 to December 19, 2021 and 872 were valid, with an effective rate of 81.12%. The total number of samples collected was conducted post hoc using the G * power 3.1.9.7 software, with an effect size d of 0.5, and a significance level of 0.05 for each sample size group of 872. Employing the two-tailed t-test, the power was infinitely close to 1.

#### Quality control

After the pre-survey test, the questionnaires that the respondents took more than 200 seconds to answer should be included in the first and second surveys, otherwise the questionnaires would be excluded. It should be noted that the screening rules in the VIP function of the “Questionnaire Star” platform combined with manual screening rules were used in the implementation of this study to exclude questionnaires that took less than 200 seconds to answer and questionnaires filled out by respondents from non-target cities. The denominator of the effective rate of questionnaires calculated in this study was the actual number of questionnaires collected after the above two conditions were excluded. The network IP address of the questionnaire was limited to the cities those were surveyed, and only respondents in these cities had access to the questionnaire. The IP address of the network should be consistent with the self-filled address. Set common sense questions as screening items, such as ambulance emergency calls. The scale also sets reverse scoring questions. Establish a unique PIN, which consists of the first letter of the respondents’ name and the last four digits of the mobile phone number. According to the basic personal information (personal identification number, IP address, gender, age, education stage) to determine whether the same person repeated filling in, the repeated questionnaires will only recognize the result of the first filling. Set up the “trap” question to see if the respondents filled in carefully. For example, a minimum score of one is required for the current doctor-patient relationship score, but the default option is set to zero, and a questionnaire with a score of zero for this item will be judged to have not been completed as required.

### Study measures

In this study, the questionnaire used a self-designed health self-consciousness scale, perceived a community with shared future for doctor-patient scale, revised 7-item generalized anxiety disorder scale and benefit finding scale. The scales distributed in the outbreak stage of COVID-19 and in the stable stage of COVID-19 are the same, which include benefit finding scale, health self-consciousness scale and perceived a community with shared future for doctor-patient scale distributed in the two periods. Only the 7-item generalized anxiety disorder scale distributed in the two periods has different situation settings. And the language expressions of the epidemic situation were added to all the items of GAD-7 (Table S1 and Table S2). Because the epidemic situation was different when the two questionnaires were sent out, the language expression of the questionnaire in the stable stage of COVID-19 was adjusted. For example, “whether I am worried that I will be infected” was adjusted to “when the epidemic will occur again in the future, do I worry that I will be infected”. The low-risk areas, medium-risk areas and high-risk areas designated by cities are divided according to the time of occurrence of cases and the impact of the epidemic, and the medium-and-high-risk areas indicate that there are new cases in the region. More than five kilometers from the nearest medium-and-high-risk area in the residential district or workplace is defined as long distance, and less than five kilometers is defined as close distance.

#### 7-Item generalized anxiety disorder scale (GAD-7)

In the early stage of the epidemic, the level of anxiety and depression in the general population increased [28]. GAD-7 is used to access anxiety. This scale is compiled by Spitzer et.al. [29]and is widely used in scientific research and clinic [29], and it is also widely used to measure the anxiety of the general population, medical staff or patients with COVID-19 in the outbreak stage of COVID-19 [28]. We have added situational restrictions to the items of the original scale to make it easier to have a sense of substitution. There are seven items in the scale (Table S1 and Table S2). The answer options are sorted in sequence: None (1 point), A few days (2 point), More than a week (3 point), Almost every day (4 point), with four-point Likert scale. According to the GAD-8 classification standard, the option “None” to “Almost every day” is reassigned from 0 to 3 when the total score of the option is calculated, with an overall score range of 0 to 21. 0 to 4 represents no anxiety, 5 to 9 indicates mild anxiety, 10 to 14 indicates moderate anxiety, and more than 15 represents severe anxiety [30]. The higher the total score of all items, the greater the anxiety. It is confirmed that the scale has good internal consistency (Cronbach’s α = 0.912).

#### Benefit finding scale (BFS)

The epidemic has shown positive benefits in increasing appreciation of life, promoting interpersonal relationships and improving health [31]. The increase in mortality caused by COVID-19 is associated with increased BF from the epidemic (such as relationship investment, gratitude, patience) [32]. The public who perceives the meaning of life will translate perceived social support into the motivation of future life [33]. As a result of the epidemic, the concept of thinking in public will change from material pursuit to inner needs, from external evaluation to inner feelings. With reference to the previous benefit subscale [34], we redesigned BFS after adding the limit of the epidemic restrictions. The redesigned BFS has three topics (Table S3). For example, one topic is “Experiencing the epidemic has made me more aware of the significance of my learning to society”. There are three items in the scale (Table S3). The answer options are sorted in sequence: Fully agree (1 point), Moderately agree (2 point), Uncertain (3 point), Moderately disagree (4 point), Fully disagree (5 point), with five-point Likert scale. All items are calculated with reverse scoring, and the scores are 5 points, 4 points, 3 points, 2 points and 1 point in turn. It is confirmed that the scale has good internal consistency (Cronbach’s α = 0.833).

#### Health self-consciousness scale (HSCS)

Traditionally, healthy lifestyles include smoking, drinking, exercise, and preventive health care behaviors (such as influenza vaccination, dental care, eye examinations) are also healthy behaviors [35]. The high-frequency spread of health science in the outbreak stage of COVID-19 has significantly enhanced the awareness of epidemic prevention and control and the reserve of medical knowledge in public [36]. COVID-19-related prevention cognition has a positive impact on healthy lifestyles [37]. Although the epidemic will make the public pay more attention to healthy life, it is more difficult to adhere to a healthy diet and participate in physical activity than to wear a mask and wash hands. Studies have confirmed that there seems to be a big difference between maintaining a healthy lifestyle and insisting on preventing infection [18]. The most obvious evidence is that the COVID-19-related prevention cognition scale is independently designed to confirm its correlation with healthy lifestyle [37], rather than being directly integrated into the healthy lifestyle scale. We believe that wearing masks and washing hands are not only subjective but also affected by external epidemics. Adhering to a healthy lifestyle is more of an internal driving force for the pursuit of health. Therefore, the items of HSCS we designed were “affected by the epidemic, now I pay more attention to personal hygiene habits” and “affected by the epidemic, now I pay more attention to a healthy lifestyle”. The epidemic makes the public deeply feel the fragility of life and the significance of health. In general, individuals with HSC know more about their health and will pay attention to their health problems, and then take health measures to ensure their health [16]. Referring to the three items of the self-consciousness subscale of health consciousness scale compiled by Gould [38], another item of the HSCS designed by us is “affected by the epidemic, now I am more concerned about my health status”. We believe that developing hygienic habits, paying attention to healthy lifestyle and giving weight to health status are all external manifestations of HSC, and there are both relevance and relative independence in the epidemic situation. There are three items in the scale (Table S4). All items of the scale were scored in reverse. The answer options are sorted in sequence: Fully agree (1 point), Moderately agree (2 point), Uncertain (3 point), Moderately disagree (4 point), Fully disagree (5 point), with five-point Likert scale. All items are calculated with reverse scoring, and the scores are 5 points, 4 points, 3 points, 2 points and 1 point in turn. It is confirmed that the scale has good internal consistency (Cronbach’s α = 0.782).

#### Perceived a community with shared future for doctor-patient scale (PCSFS)

The global COVID-19 confirms that relationships around the world are interdependent [39]. To effectively deal with the spread of the virus, a community with shared future for mankind has been advocated [2]. Life safety and physical health have become the only goal of both doctors and patients [40], and the epidemic has rapidly sublimated the doctor-patient relationship into a community with shared future for doctor-patient. The common enemy of all mankind is COVID-19, not the infected person [2]. Analogy to the field of doctor-patient relationship, the common enemy of medical staff and patients should be disease. Therefore, one of the items of the PCSFS is “experiencing the epidemic has made me realize more deeply that the common enemy between doctors and patients is disease”. When treating patients with COVID-19, Chinese health care workers have shown great professional dedication and voluntarily put themselves in danger of overworking [3]. Studies have shown that respondents in the second survey have strong confidence in the ability of doctors to diagnose or recognize COVID-19 and have better chance of survival in comparison with respondents in the first survey [41]. Trust of patients in medical staff can increase the motivation of medical staff to treat the disease, and the due diligence of medical staff towards patients will increase the courage of patients to overcome the disease. Therefore, one of the items of the PCSFS is “experiencing the epidemic has made me realize more deeply that coping with the disease requires the joint efforts of doctors and patients”. A survey in the outbreak stage of COVID-19 found that the public perceived the limitations of extensive public health education and modern medicine as the third factor in improving doctor-patient relations (a total of nine options) [42]. The outbreak made the public aware of the limitations of modern medicine and began to sympathize with and support medical staff. In addition to outbreaks of infective diseases, the response to chronic diseases is not optimistic. It is impossible to deal with these diseases completely by relying on medical science and technology alone [43]. Therefore, another item of the PCSFS is “experiencing the epidemic has made me more aware of the limitations of modern medical technology”. There are three items in the scale (Table S5). The answer options are sorted in sequence: Fully agree (1 point), Moderately agree (2 point), Uncertain (3 point), Moderately disagree (4 point), Fully disagree (5 point), with five-point Likert scale. All items are calculated with reverse scoring, and the scores are 5 points, 4 points, 3 points, 2 points and 1 point in turn. It is confirmed that the scale has good internal consistency (Cronbach’s α = 0.868).

### Statistical analysis

Propensity score matching takes the tendency score as the matching condition, and the subjects with the same or similar tendency score are matched according to the proportion of 1:1 or 1: N to obtain a baseline comparable sub-database, and then analyze the relationship between independent variables and dependent variables. Before statistical analysis, we use propensity score matching to match the two samples in the outbreak stage of COVID-19 and in the stable stage of COVID-19. Matching tolerance (caliper value) refers to the accuracy of the match, the value range is zero to one, the closer to zero, the more accurate, the closer to one, the more blurred the match. We set the caliper value to 0.02. We chose the sampling method that does not put back and give priority to an exact match. Chi-square test was used to compare the difference of the overall frequency (constituent ratio) of the confounding factors between the two samples before and after the propensity score matching, so as to evaluate whether the distribution of confounding variables was balanced between the two groups.

Using SPSS to establish moderating model and mediating model requires tedious steps, and multiple models can only be tested by segments. Therefore, to solve this malpractice, Hayes scholars have developed a free plug-in PROCESS that can be applied to SPSS to assist researchers in directly analyzing the model which includes mediating effects, moderating effects, or both [44]. PROCESS is a percentile Bootstrap method based on deviation correction. In addition to the results of conventional regression analysis, PROCESS also provides the estimated values of direct and indirect effects, Bootstrap confidence interval (CI), Sobel test and so on. We use PROCESS 3.5 version of the seven model. The simple slope was tested in the case of M ± 1SD.

Considering that the expression of the scale has been adjusted or redesigned, we use exploratory factor analysis to explore the dimension division of the scale and use reliability analysis to test the consistency or reliability of the results. The continuous variables are tested by P-P plot and histogram to see if they obey normal distribution. Count data are expressed as examples and percentages. The use of independent-sample t-tests on two sets of data after propensity score matching is very common in previous literature [45]. For uniformly distributed data, the efficiency of the t-test and rank sum test is the same. However, for the data of skewed distribution, the function of the t-test is not as good as that of rank sum test, and the advantage of rank sum test is more obvious. When the normal distribution and homogeneity of variance can’t meet the requirements of t-test, we use Mann-Whitney U test to compare the two groups. When multiple groups of samples do not satisfy the hypothesis of normal distribution and homogeneity of variance, we use Kruskal-Wallis to test whether multiple groups of samples come from the same population, and use Bonferroni method to correct the pairwise comparison of significance level. At the same time, the median is used for statistical description. Spearman rank correlation test is used to analyze the correlation between two variables that do not satisfy normal distribution. Tested by histogram and P-P plot, the total scores of HSC, PCSF and BF in the outbreak stage of COVID-19 and anxiety, HSC, PCSF and BF in the stable stage of COVID-19 showed skewed distribution, while the total score of anxiety showed approximate normal distribution in the outbreak stage of COVID-19. When comparing the two groups of data, one group of data does not satisfy the normality, so the rank sum test is given priority.

## Results

### Propensity score matching

Before matching, the median age was 30 years old in the outbreak stage of COVID-19 and 32 years old in the stable stage of COVID-19. The median age of all samples was 31 years old. Therefore, the age of matching is set at 31 years old (people younger than thirty-one years old present zero, the rest present one). The exposure factor of this study was the epidemic situation (in the outbreak stage of COVID-19 and in the stable stage of COVID-19), and the outcomes included PCSF, HSC and BF. Confounding factors may include age, gender, education level, marriage and distance from medium-to-high risk areas. Match in a one-to-one ratio, there are 795 accurate matching cases, 30 fuzzy matching cases and 47 unsuccessful matching cases.

**Fig. 1:**
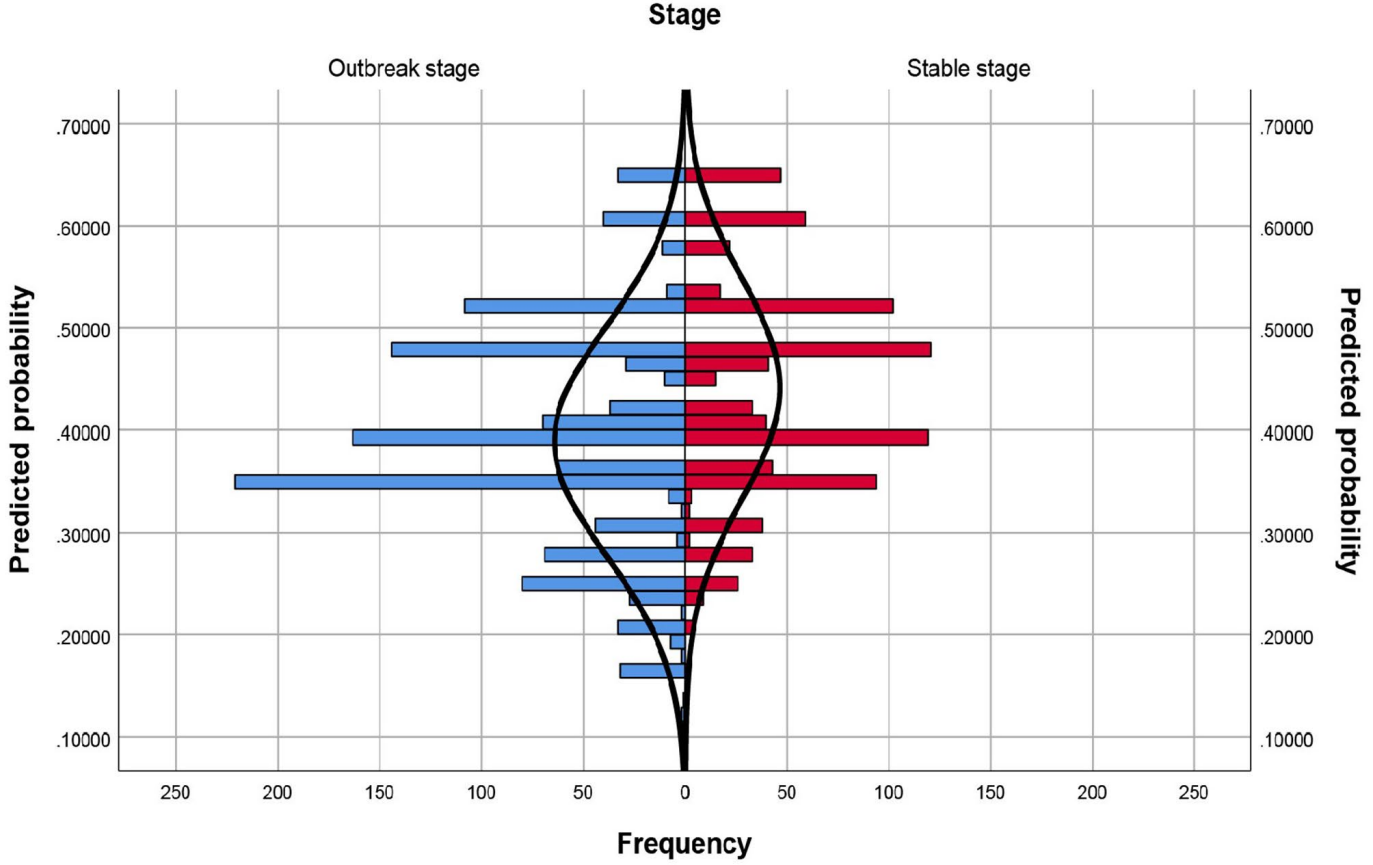
Population pyramid chart before matched

The population pyramid chart before matching showed that the distribution of propensity score was significantly different in the outbreak stage of COVID-19 and in the stable stage of COVID-19. (Fig. 1)

**Fig. 2:**
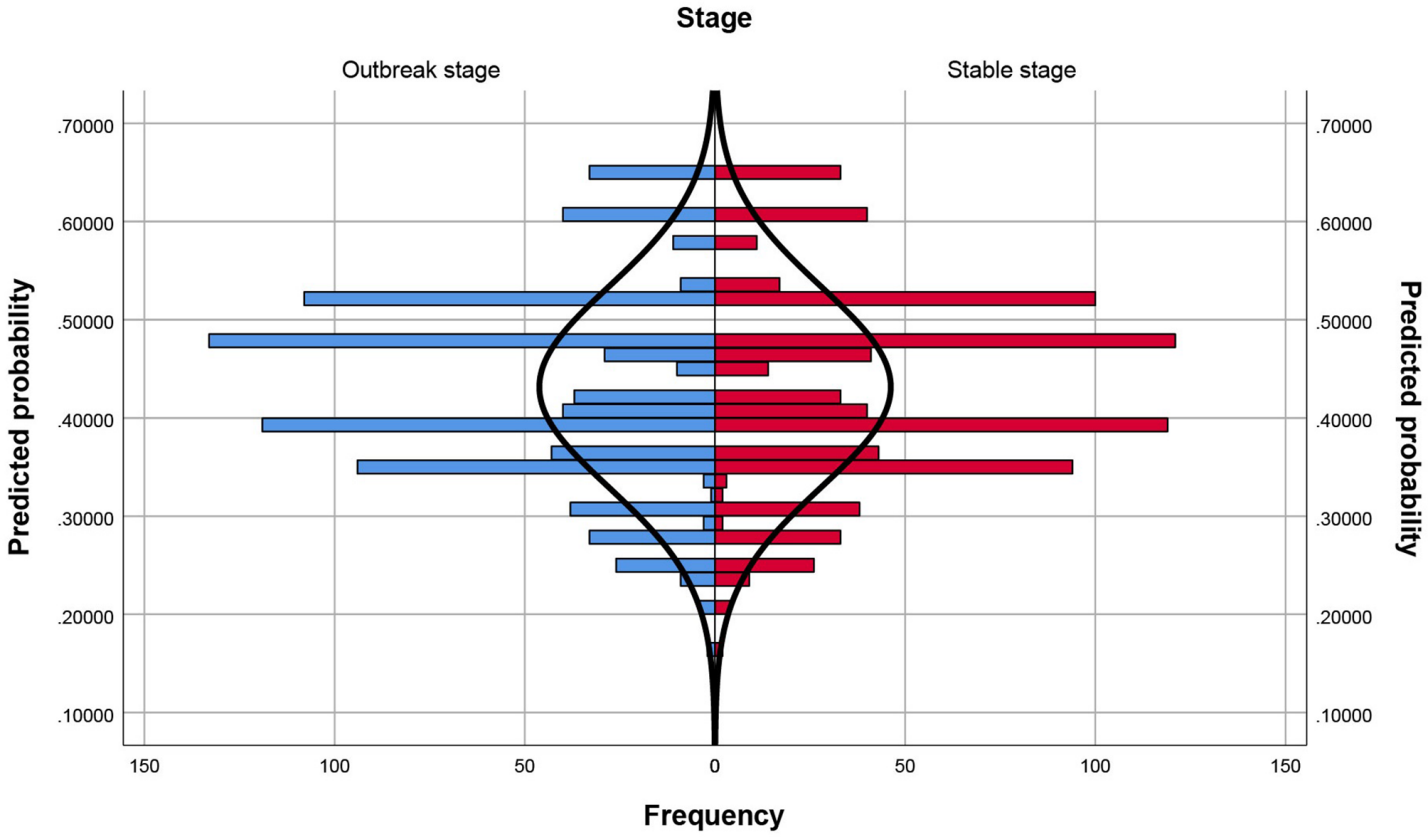
Population pyramid chart after matched

The population pyramid chart after matching shows that the propensity score has been basically balanced between the two groups, which proves that this matching is more successful. (Fig. 2)

Before matching, except for gender (χ^2^ = 2.715, *P* = .099), there were significant differences in age (χ^2^ = 41.597, *P* < .001), education level (χ^2^ = 19.310, *P* < .001), marriage (χ^2^ = 15.713, *P* < .001) and distance from medium-and-high-risk areas (χ^2^ = 15.285, *P* < .001) in the outbreak stage of COVID-19 and in the stable stage of COVID-19. After matching, it was comparable between age (χ^2^ = 0.161, *P* = .689), education level (χ^2^ = 0.092, *P* = .955), gender (χ^2^ = 0.196, *P* = .658), marriage (χ^2^ = 0.422, *P* = .516), distance from medium-to-high-risk area (χ^2^ = 0.346, *P* = .556) in the outbreak stage of COVID-19 and in the stable stage of COVID-19. Generally speaking, the distribution of mixed variables between the two groups is uneven before matching, and is balanced after matching. (Table 1)

**Table 1:** Comparison of demographic characteristics among respondents after propensity score matching

	In the outbreak stage of COVID-19 N (%)	In the stable stage of COVID-19 N (%)	χ^2^	*P*
**Age group(years)**				
<31	332(40.2)	340(41.2)	0.161	0.689
≥ 31	493(59.8)	485(58.8)		
**Gender**				
Male	418(50.7)	409(49.6)	0.196	0.658
Female	407(49.3)	416(50.4)		
**Marital status**				
Married	590(71.5)	578(70.1)	0.422	0.516
Unmarried	235(28.5)	247(29.9)		
**Education**				
Senior high school (technical secondary school) and below	349(42.3)	355(43.0)	0.092	0.955
Junior college	372(45.1)	368(44.6)		
Bachelor degree or above	104(12.6)	102(12.4)		
**Distance**				
Long distance	715(86.7)	723(87.6)	0.346	0.556
Close distance	110(13.3)	102(12.4)		

### Reliability and validity analysis

The method of maximum rotation of variance was used for exploratory factor analysis. In the outbreak stage of COVID-19, the KMO values of anxiety, HSC, PCSF and BF are 0.925, 0.680, 0.736 and 0.726 respectively. In the stable stage of COVID-19, the KMO values of anxiety, HSC, PCSF and BF were 0.901, 0.722, 0.747 and 0.702 respectively. There was one common factor with a characteristic value greater than one and the explanatory variation was more than 50% through validity analysis in all scales.

In the outbreak stage of COVID-19, the Cronbach’s α of anxiety was 0.912, the Cronbach’s α of HSC was 0.782, the Cronbach’s α of PCSF was 0.868, and the Cronbach’s α of BF was 0.833. In the stable stage of COVID-19, the Cronbach’s α of anxiety was 0.868, the Cronbach’s α of HSC was 0.835, the Cronbach’s α of PCSF was 0.891, and the Cronbach’s α of BF was 0.801. All the items of the scales have a good degree of consistency.

### Factors of anxiety and PCSF in the outbreak stage of COVID-19 and in the stable stage of COVID-19

Factors that may cause infection may increase anxiety in public, while related personal preventive measures may be protective factors of anxiety [46]. We speculate that even if there is no actual exposure risk but just a premonition of the risk will increase anxiety, and relying solely on personal prevention may have limited effect. Urban prevention and control measures can provide the public with a strong external sense of security. In the stable stage of COVID-19, the proportion of citizens worried about infection decreased (χ^2^ = 70.757, *P* < .001), the proportion of citizens who actively paid attention to the epidemic decreased (χ^2^ = 511.629, *P* < .001), and the proportion of citizens with growing trust in medical staff increased (χ^2^ = 3.906, *P* = .048). In the outbreak stage of COVID-19 and in the stable stage of COVID-19, the proportion of citizens who trusted the ability of the government to prevent and control the epidemic was 97.2% and 96.4% respectively, and the degree of trust in the ability of the government was always at a high level, and there was no statistically significant difference between them (χ^2^ = 0.955, *P* = .328).

According to the evaluation and grading standard of GAD-7, the total score of the scale ranged from zero to twenty-one. The score of zero-to-four means no anxiety. The score of five-to-nine means mild anxiety. The score of ten-to-fourteen means moderate anxiety, and greater than or equal to fifteen points means severe anxiety [30]. In the outbreak stage of COVID-19, 134 people were not anxious (16.2%), 238 people were mildly anxious (28.8%), 272 people were moderately anxious (33.0%), and 181 people were severely anxious (21.9%). In the stable stage of COVID-19, 506 people had no anxiety (61.3%), 236 people had mild anxiety (28.6%), 81 people had moderate anxiety (9.8%), and 2 people had severe anxiety (0.2%). In the stable stage of COVID-19, the proportion of people with moderate and severe anxiety decreased significantly (χ^2^ = 494.666, *P* < .001).

Mann-Whitney U test showed that people who were worried about being infected were more likely to be anxious than those who were not worried about infection in the outbreak stage of COVID-19 (Z = -8.626, *P* < .001) and in the stable stage of COVID-19 (Z = -4.428, *P* < .001). Kruskal-Wallis H test showed that the distribution of anxiety of people with different attitudes towards the epidemic was not the same in the outbreak stage of COVID-19 (H = 34.956, *P* < .001) and in the stable stage of COVID-19 (H = 27.292, *P* < .001), and the difference was statistically significant. After the pairwise comparison of the significance level corrected by Bonferroni method, it was found that there was significant difference in the distribution of anxiety between the active concern group and the non-concern group, and between the active concern group and the passive concern group (adjusted *P* = .006 and *P* < .001), but there was no significant difference between the non-concern group and the passive concern group. Combined with the average rank, it is suggested that those who actively pay attention to the epidemic situation have the highest anxiety in the outbreak stage of COVID-19. In the stable stage of COVID-19, there were significant differences in the distribution of anxiety between active concern group and passive concern group, and between active concern group and non-concern group (adjusted *P* < .001 and *P* = .001). There were significant difference in the distribution of anxiety between passive concern group and non-concern group (adjusted *P* = .006). Combined with the average rank, it is suggested that those who actively pay attention to the epidemic situation have the highest anxiety in the stable stage of COVID-19, followed by those who passively pay attention to the epidemic situation, and finally those who do not pay attention to the epidemic situation. Hypothesis one (H1) is verified.

Medical staff are important participants in the PCSF, which will directly determine the level of the PCSF in public. The government is the main body of medical policy-making and the driving force of a community with shared future for doctor-patient. The intimacy of cooperation between the public and medical staff also reflects the level of the support and trust of the public in government policies. In the outbreak stage of COVID-19 and in the stable stage of COVID-19, the probability of the PCSF in the citizens who had increased trust in medical staff was higher than that in those who did not improve their trust in medical staff (Z = -7.297, *P* < .001 and Z = -4.314, *P* < .001). In the outbreak stage of COVID-19, the probability of PCSF in the citizens who trusted the prevention and control ability of the government was higher than that in those who did not trust prevention and control ability of the government (Z = -4.846, *P* < .001). In the stable stage of COVID-19, there was no significant difference of the probability of PCSF between citizens who trusted the prevention and control ability of the government and those who did not trusted prevention and control ability of the government (Z = -1.649, *P* = .099). Hypothesis two (H2) is partially verified.

### Changes in anxiety, HSC, PCSF and BF

By histogram and P-P plot test, the total scores of HSC, PCFS, BF and GAD-7 in the stable stage of COVID-19 were skewed, and the total scores of GAD-7 were approximately normal in the outbreak stage of COVID-19. The results of Mann-Whitney U test showed that there were significant differences in anxiety (Z = -22.979, *P* < .001), HSC (Z = -6.640, *P* < .001) and BF (Z = -6.874, *P* < .001) in the outbreak stage of COVID-19 and in the stable stage of COVID-19. There was no significant difference in PCSF in the outbreak stage of COVID-19 and in the stable stage of COVID-19 (Z = -0.066, *P* = .947). Combined with median value and average rank, anxiety, HSC and BF decreased in the stable stage of COVID-19, while there was no significant change in PCSF. Hypothesis three (H3) is partially verified. (Table 2)

**Table 2:** Changes in anxiety, health self-consciousness, perceived a community with shared future for doctor-patient, and benefit finding*

	Anxiety	HSC	PCSF	BF
In the outbreak stage M (P_25_, P_75_)	10.00 (6.00, 14.00)	12.00 (10.00, 14.00)	11.00 (9.00, 13.00)	11.00 (10.00, 13.00)
In the stable stage M (P_25_, P_75_)	3.00 (1.00, 7.00)	11.00 (8.00, 13.00)	11.00 (8.00, 13.00)	10.00 (8.00, 12.00)
Z	-22.979	-6.640	-0.066	-6.874
*P*	<0.001	<0.001	0.947	<0.001

* HSC: Health Self-Consciousness; PCSF: Perceived a Community with Shared Future for Doctor-Patient; BF: Benefit finding

### Correlation among anxiety, HSC, PCSF and BF

The total scores of anxiety, HSC, PCSF, and BF were standardized. Spearman correlation analysis showed that there was a positive correlation among anxiety, HSC, PCSF and BF in the outbreak stage of COVID-19 (*P* < .001). In the stable stage of COVID-19, anxiety was positively correlated with HSC (*P* < .001) and PCSF (*P* = .001), but there was no significant correlation between anxiety and BF (*P* = .067). There was a positive correlation among HSC, PCSF and BF (*P* < .001). In the stable stage of COVID-19, the majority of people had no anxiety and mild anxiety in the case of the removal of risk factors. The correlation between anxiety and other factors decreased. Hypothesis four (H4) is partially verified. (Table 3)

**Table 3:** Correlation analysis of anxiety, health self-consciousness, perceived a community with shared future for doctor-patient, and benefit finding*

		Anxiety	HSC	PCSF
In the outbreak stage	Anxiety	1.000	0.352^**^	0.152^**^
	HSC	0.352^**^	1.000	0.219^**^
	BF	0.274^**^	0.308^**^	0.496^**^
In the stable stage	Anxiety	1.000	0.269^**^	0.111^**^
	HSC	0.269^**^	1.000	0.135^**^
	BF	0.064	0.191^**^	0.348^**^

^**^:*P* < .01

*HSC: Health Self-Consciousness; PCSF: Perceived a Community with Shared Future for Doctor-Patient; BF: Benefit finding

### Mediating effect of HSC between PCSF and BF

With the increase of age and life experience, individuals usually constantly revise their values in social practice. In most cases, there are differences in the way of men and women think, which also leads to their different feelings about the same thing. Generally speaking, the higher the level of education, the higher the cognitive level, and the process of education is also the process of improving cognitive ability. The most important feature of marriage is the formation of a family. Married people not only shoulder the responsibility of the family but also have the advantage of being shared by the pressure. Therefore, the values and emotional adjustment ability of married people may be different from those of unmarried people. The distance between the individual and the medium-and-high-risk areas will determine whether to feel the epidemic directly or receive the epidemic information indirectly. People in different situations will have different degrees of emotional reactions. Therefore, we set age, gender, education level, marriage and distance from medium-to-high-risk areas as control variables.

After matching, with reference to bachelor’s degree or above, virtual variables are set for the education level. PCSF is set as an independent variable, HSC as a mediating variable, anxiety as a moderator variable and BF as a dependent variable. The seven model of PROCESS that plugs in SPSS was used to test the mediating role of HSC in the influence of PCSF on BF and the moderator role of anxiety in the influence of PCSF on HSC.

When BF was the dependent variable, the R values of the model in the outbreak stage of COVID-19 and in the stable stage of COVID-19 were 0.555 and 0.429, the R^2^ values were 0.308 and 0.184, and *P* values were less than .001, which indicated that the two models were statistically significant. The results of mediating effect test showed that the confidence intervals were 0.3785 to 0.5007 and 0.2357 to 0.3695 respectively, indicating that some mediating effects existed (*P* < .001). PCSF had direct positive effects on the BF in the outbreak stage of COVID-19 or in the stable stage of COVID-19, it also indirectly affected BF through HSC. Hypothesis five (H5) is verified. (Table 4)

**Table 4:** A test of the mediating effect of health self-consciousness between perceived a community with shared future for doctor-patient and benefit finding*

		Unstandardized Coefficients		t	*P*	95% Confidence Interval	
		B	Std. Error			Lower Bound	Upper Bound
In the outbreak stage of COVID-19	Constant	-0.415	0.289	-1.437	0.151	-0.983	0.152
	PCSF	0.440	0.031	14.121	< 0.001	0.378	0.501
	HSC	0.182	0.033	5.586	< 0.001	0.118	0.246
	Gender	-0.001	0.059	-0.016	0.987	-0.116	0.114
	Marriage	-0.012	0.086	-0.141	0.888	-0.180	0.156
	Distance	0.135	0.088	1.544	0.123	-0.037	0.307
	Senior high school (technical secondary school) and below	0.102	0.104	0.986	0.325	-0.102	0.307
	Junior college	0.220	0.092	2.388	0.017	0.039	0.401
	Age	0.012	0.008	1.561	0.119	-0.003	0.028
In the stable stage of COVID-19	Constant	-0.426	0.345	-1.235	0.217	-1.102	0.251
	PCSF	0.303	0.034	8.879	< 0.001	0.236	0.369
	HSC	0.151	0.032	4.677	< 0.001	0.088	0.214
	Gender	-0.013	0.065	-0.198	0.843	-0.141	0.115
	Marriage	-0.364	0.085	-4.262	< 0.001	-0.532	-0.196
	Distance	-0.083	0.101	-0.820	0.413	-0.281	0.116
	Senior high school (technical secondary school) and below	0.261	0.121	2.164	0.031	0.024	0.498
	Junior college	0.042	0.103	0.403	0.687	-0.161	0.245
	Age	0.009	0.009	0.953	0.341	-0.009	0.027

*HSC: Health Self-Consciousness; PCSF: Perceived a Community with Shared Future for Doctor-Patient

### The moderating effect of anxiety between PCSF and HSC

**Fig. 3:**
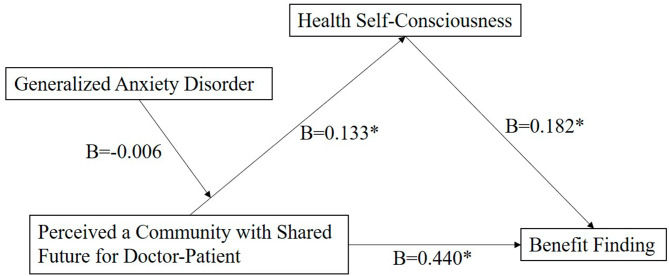
Path model examining the moderated mediation effect among BF, HSC, PCSF and GAD in the outbreak stage of COVID-19. BF = Benefit Finding; HSC = Health Self-Consciousness; GAD = Generalized Anxiety Disorder; PCSF = Perceived a Community with Shared Future for Doctor-Patient; B means unstandardized coefficients of paths; *P < .05.

**Fig. 4:**
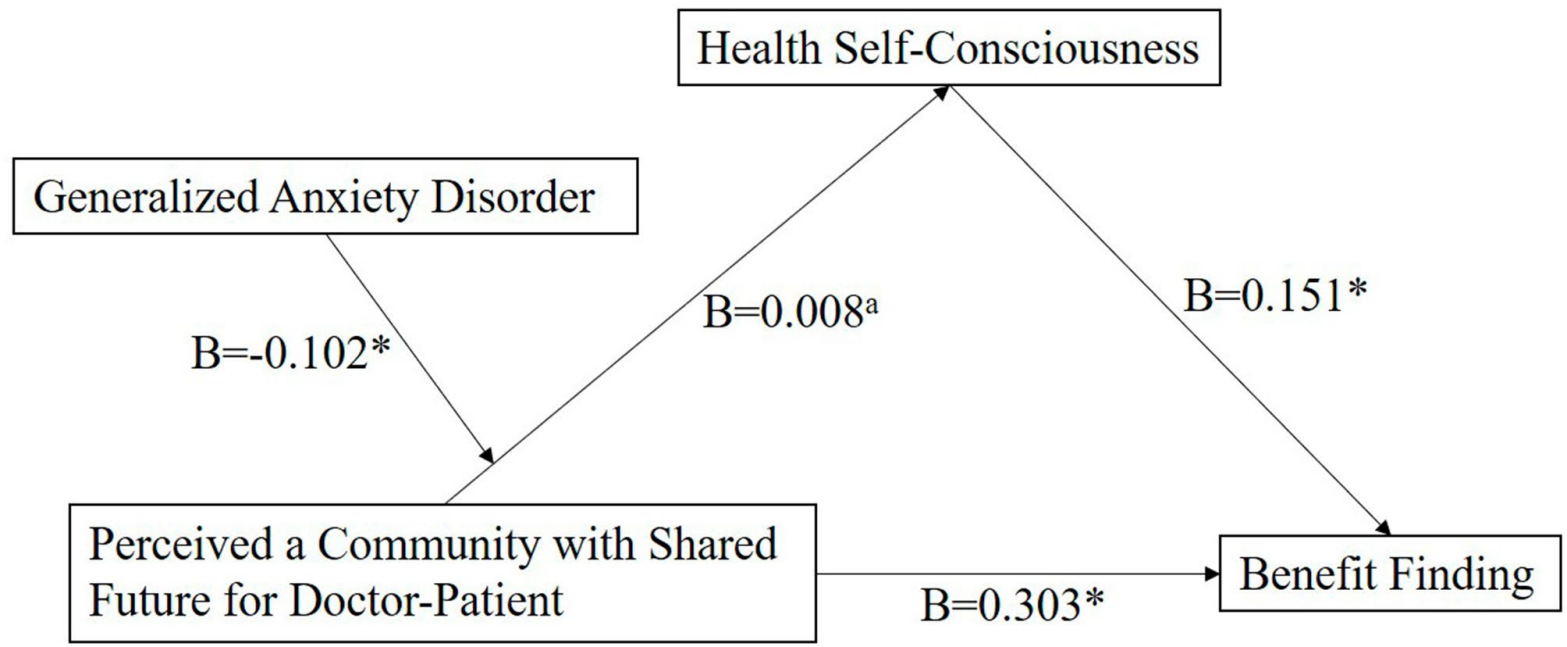
Path model examining the moderated mediation effect among BF, HSC, PCSF and GAD in the stable stage of COVID-19. BF = Benefit Finding; HSC = Health Self-Consciousness; GAD = Generalized Anxiety Disorder; PCSF = Perceived a Community with Shared Future for Doctor-Patient; B means unstandardized coefficients of paths; *P < .05?^a^: The main effect was significant before introducing the moderating effect (anxiety ✕ PCSF).

When HSC was the dependent variable, the R values of the model in the outbreak stage of COVID-19 and in the stable stage of COVID-19 were 0.432 and 0.402, R^2^ values were 0.187 and 0.161 respectively (*P* < .001), which indicated that the two models were statistically significant. PCSF will positively affect HSC. However, in the outbreak stage of COVID-19, although anxiety will positively affect HSC, the moderating effect of anxiety between PCSF and HSC does not exist (*P* = .840). Hypothesis six (H6) is partially verified. In the stable stage of COVID-19, the main effect was significant before introducing the moderating effect (anxiety ✕ PCSF). After introducing the moderating effect, the coefficient of anxiety ✕ PCSF was negative, the B values of anxiety and PCSF were positive, and the moderating effect was negative (*P* = .038). Anxiety had a negative moderating effect on PCSF and HSC, which indicated that anxiety would weaken the effect of PCSF on HSC. In terms of HSC, as anxiety falls, PCSF, which is called the substitution relationship. Hypothesis six (H6) is verified. (Table 5). According to the research data above, charts of the path model are created to describe the moderated mediation effect of anxiety between HSC and PCSF in the outbreak stage of COVID-19 and in the stable stage of COVID-19. (Figures 3 and 4)

**Table 5:** A test of the moderating effect of anxiety between perceived a community with shared future for doctor-patient and health self-consciousness*

		Unstandardized Coefficients		t	*P*	95% Confidence Interval	
		B	Std. Error			Lower Bound	Upper Bound
In the outbreak stage	Constant	-0.925	0.300	-3.080	0.002	-1.514	-0.335
	PCSF	0.133	0.036	3.741	<0.001	0.063	0.203
	Anxiety	0.254	0.033	7.682	<0.001	0.189	0.319
	PCSF ✕ anxiety	-0.006	0.030	-0.202	0.840	-0.064	0.052
	Gender	0.142	0.061	2.336	0.020	0.023	0.261
	Marriage	0.031	0.089	0.349	0.727	-0.144	0.206
	Distance	0.446	0.091	4.903	<0.001	0.268	0.625
	Senior high school (technical secondary school) and below	0.009	0.108	0.084	0.933	-0.203	0.221
	Junior college	0.002	0.096	0.017	0.987	-0.186	0.189
	Age	0.026	0.008	3.118	0.002	0.010	0.042
In the stable stage	Constant	-0.931	0.363	-2.562	0.011	-1.644	-0.218
	PCSF	0.008	0.047	0.164	0.870	-0.084	0.099
	Anxiety	0.327	0.050	6.507	<0.001	0.229	0.426
	PCSF ✕ anxiety	-0.102	0.049	-2.078	0.038	-0.198	-0.006
	Gender	0.309	0.068	4.513	<0.001	0.175	0.443
	Marriage	-0.118	0.090	-1.305	0.192	-0.295	0.059
	Distance	0.410	0.108	3.809	<0.001	0.199	0.621
	Senior high school (technical secondary school) and below	-0.119	0.127	-0.939	0.348	-0.369	0.130
	Junior college	-0.146	0.109	-1.337	0.182	-0.360	0.068
	Age	0.028	0.010	2.881	0.004	0.009	0.047

*HSC: Health Self-Consciousness; PCSF: Perceived a Community with Shared Future for Doctor-Patient

**Fig. 5:**
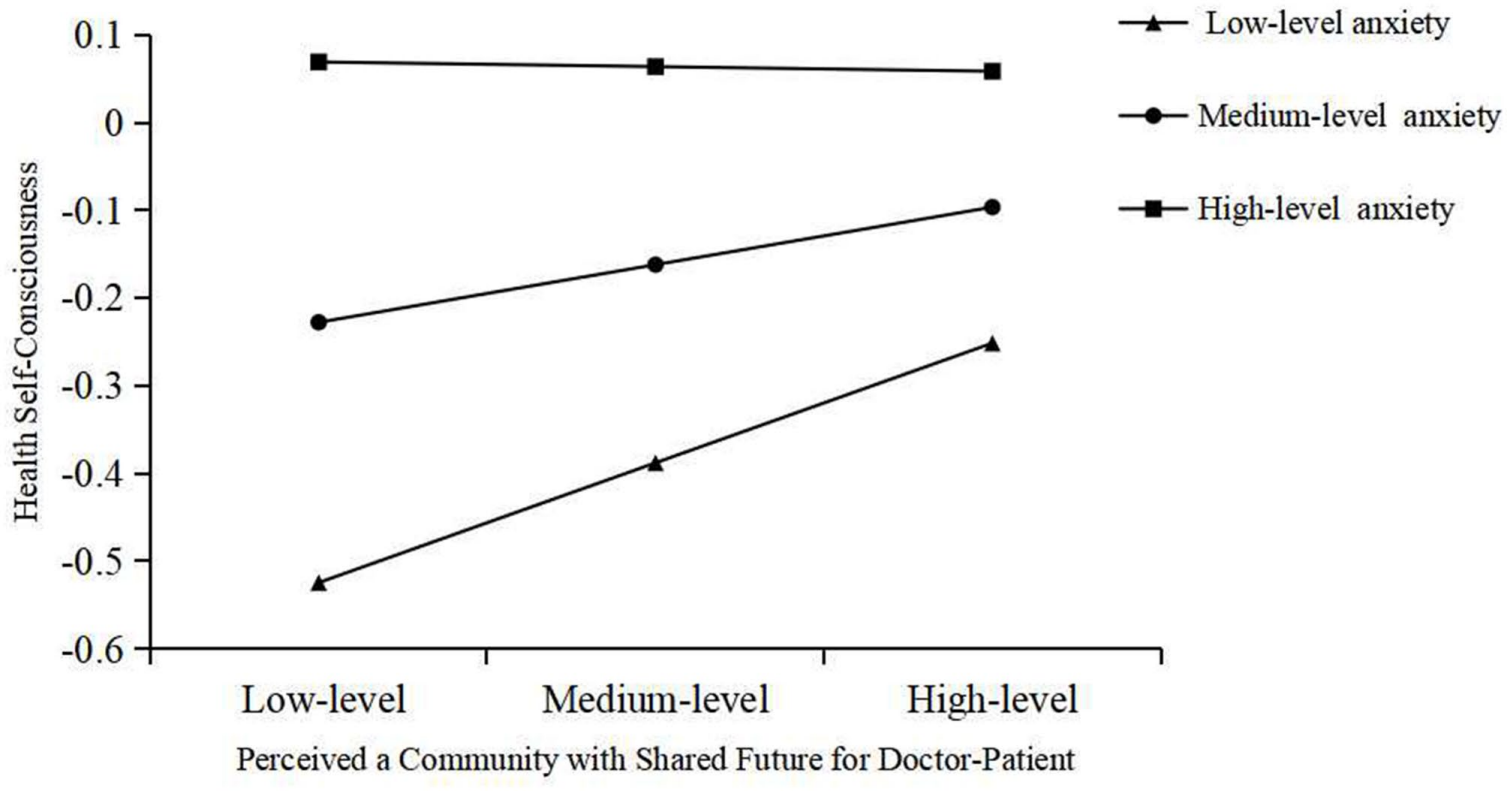
Simple slope plot of moderating effect

We carried out a simple slope test on the moderating effect in the stable stage of COVID-19. At the low level of the moderator variable (M-1SD), the *P* value of the test is significant (*P* = .0050, 95% CI = [0.0409, 0.2296]), and the upper and lower limits of the confidence interval do not include zero, which indicates that the moderating effect of the moderator variable at the low level is significant, and the simple slope is 0.1353, which has a positive impact on the mediating variable as the dependent variable. At the middle level (M) of the moderator variable, the *P* value of the test is not significant (*P* = .0716, 95% CI = [-0.0057, 0.1358]), and the upper and lower limits of the confidence interval included zero, indicating that the moderating effect is not significant at this time, with a simple slope of 0.0650. At the high level of the moderator variable (M + 1SD), the *P* value of the test is not significant (*P* = .9178, 95% CI = [-0.1049, 0.0944]). The upper and lower limits of the confidence interval include zero, indicating that the moderating effect is not significant, and the simple slope is -0.0052. In short, moderator variables only have effects on mediating variables at low levels, and the moderating effect of moderator variables between independent variables and mediating variables is established. Hypothesis six (H6) is verified again. (Fig. 5)

## Discussion

This research is a longitudinal study of adults that are not medical staff to investigate the possible correlation between anxiety, BF, HSC and PCSF in the context of the epidemic in China. This study collected the changes of the public’s psychological state in the outbreak stage of COVID-19 and in the stable stage of COVID-19 in China, which has a certain guiding significance for people’s psychological health intervention and post-traumatic psychological health improvement.

A community with shared future for doctor-patient can be explained by the common intra-group identity model, that is, through re-classification to construct a higher identity to weaken the intergroup boundary [47]. According to the broad definition of both medical staff and patients, the public may become potential patients. The recognition of a community with shared future for doctor-patient begins with the epidemic, but it will not be limited to dealing with the epidemic. Even in epidemic control, the concept of PCSF is also applicable to other diseases. Therefore, the persistence of PCSF exists in the development of the epidemic. Chinese medical staff showed great professionalism in the process of dealing with the epidemic [48]. Compliance of the patients increased, and mutual trust and respect between medical staff and patients increased [49]. The result has shown that PCSF is affected by the trust of the public in medical staff and patients and the ability of the government in epidemic prevention and control in the outbreak stage of COVID-19 (H2). Unity and mutual assistance are in the common interests of all mankind [36]. In the outbreak stage of COVID-19, the common goal of both medical staff and patients is to fight against COVID-19. The direct participation and effective actions of the public in the outbreak stage of COVID-19 have played a positive role in promoting a harmonious doctor-patient relationship. The core of social relations is cooperation. Cooperation is conducive to the well-being of the people [1].

Anxiety is a psychological stress in the context of the epidemic. Compared with the situation in the outbreak stage of COVID-19, the correlation between the anxiety of most respondents and the epidemic weakened in the stable stage of COVID-19. Of course, a small number of respondents were still immersed in anxiety about previous outbreaks. Therefore, we believe that the removal of the risk factor of the epidemic is equivalent to a more obvious distinction between people with high and low levels of anxiety, which means that there is heterogeneity between them. Excessive protective behavior can aggravate public anxiety [50]. In the outbreak stage of COVID-19, the spread of COVID-19 was unknown, and those who worry about being infected and actively pay attention to the epidemic situation have the highest probability of anxiety. Those actively concerned about the epidemic in the outbreak stage of COVID-19 and in the stable stage of COVID-19 have the highest anxiety (H1). A study in Belgium also pointed out that risk perception and general anxiety were positively correlated in the outbreak stage of COVID-19 [51]. Compared with those in the outbreak stage of COVID-19, the anxiety, PCSF, HSC and BF decreased in the stable stage of COVID-19 (H3). So, in the stable stage of COVID-19, the risk of COVID-19 infection was relieved, and the proportion of people who worried about being infected and actively paid attention to the epidemic situation decreases so does the public anxiety. This confirms that HSC is affected by anxiety caused by perceived risk. Reduced perceived risk will also reduce HSC of protection and behavior, such as hygiene habits may return to laziness, diet and rest return to irregularity, and so on. The mental and psychological troubles of the public often come from uncertainty. The inner desire centered on interpersonal relationship and self-acceptance may be an important mechanism for people to choose to pursue the meaning of life in uncertain life events [52].

On the contrary, a high level of anxiety can also make individuals pay too much attention to their health. The occurrence of uncertain events may stimulate the public to discuss and reflect on the social phenomena caused by the events. It has been found that there was correlation among anxiety, PCSF, HSC and BF (H4). In the stable stage of COVID-19, there was a negative moderating effect of anxiety between PCSF and HSC (H6). The stronger the anxiety, the lower the positive role of PCSF in HSC. When anxiety reaches a high level, anxiety will replace the influence of other factors on HSC. This explains the substitution relationship between anxiety and PCSF (H6). This would also suggest that without the influence of uncertain events, perception and reflection will be reduced, and BF will be reduced. Studies have confirmed that survivors of COVID-19 are at risk of mental sequelae, but symptoms usually will be improved over time [53]. In the stable stage of COVID-19, the recovery of some members’ mental health in public lagged behind. In addition to overly healthy behavior, it needs to be alert whether these people have other extreme behaviors.

Although reminders of death can be negative and defensive, it can also lead people to positive life trajectories and beneficial outcomes (such as forgiveness, help) [32]. HSC, PCSF and BF are all the results of spiritual benefit inspired by negative experience. The transformation results of these spiritual benefits are stable, long-term and related. These spiritual benefits are likely to be maintained even in the stable stage of COVID-19. The epidemic is the common enemy of all mankind [2]. It is in the common interest of all mankind to unite and help each other to fight against the COVID-19 epidemic [36]. Medical staff are the most important force in preventing the spread of COVID-19 and protecting public health [54]. They have made significant contributions to controlling the spread of the epidemic [2]. With the further development of the fight against the epidemic, the health awareness of patients has gradually improved. The direct participation of patients has promoted the decision-making between medical staff and patients, improved the trust between medical staff and patients, and promoted the harmonious development of the doctor-patient relationship [55]. PCSF both directly and positively influences BF and indirectly through HSC (H5). The relationship between individuals and egos, others and society effectively enhance the sense of meaning by meeting the needs of connections between individual psychological levels. In addition, building PCSF is an important strategy for both medical staff and patients to overcome disease and maintain health, and only with health is it possible to plan a more meaningful life. In short, the common goal of medical staff and patients is health, and health is the premise of the meaning of life. This explains the mediating role of HSC. The life value of patients and the significance of medical staff are gradually realized and tested in medical practice. Vigorously advocating the concept of a community with shared future for doctor-patient can establish a good sense of HSC and enhance the understanding of the meaning of life.

### Limitation

The convenient sampling method and online survey method used in this paper may lead to selection deviation, and the representativeness of the sample is limited. The results of the study may not reflect the attitude of the whole population. Future research subjects should be expanded to cover participants of other ages. In this study, a quantitative study was used, and the attitude of the participants was reflected by the score of the scale. Further research can consider the combination of quantitative interviews and qualitative interviews. For example, adding in-depth interviews can help us understand the impact of the epidemic on the meaning of life. This study explored the mediating role of HSC between PCSF and BF. However, there may be other mediating effects of PCSF on BF. Although the reliability and validity of the scale are good, the setting of the question is simple. In the future, a multi-dimensional scale should be constructed according to the special situation of the epidemic to measure more accurately.

### Implications to research, policy, and practice

Anxiety, PCSF, HSC and BF are closely related to the occurrence and development of the epidemic. This research is the first to describe the relationship of these four variables in the context of the epidemic in China. In the outbreak stage of COVID-19, the common goal of medical staff and patients is to fight against COVID-19. In this process, the public has not only improved HSC, but also had a further understanding and feeling of the meaning of life. Direct public participation and effective action in the outbreak stage of COVID-19 also promote harmonious doctor-patient relationship. The construction of a community with shared future for doctor-patient needs multi-level protection. For example, meliorate the reform of the medical system, achieve a balanced distribution of medical resources, enhance the humanistic literacy of medical staff, ameliorate empathy between medical staff and patients, strengthen the positive guidance of public opinion, and improve the health literacy of the public. In addition, psychological intervention should not be restricted in the outbreak stage of COVID-19, but should be maintained for some time or individualized psychological intervention. In the long run, establishing meaning will help those who suffer from this epidemic live a better life [56]. For individuals who have experienced trauma before, the superposition effect of the epidemic will cause more complex psychological trauma. Comprehensive meaning therapy is proposed as the future of psychotherapy [57]. We support the coordination of existential psychology and positive psychology to achieve a better intervention effect.

## Conclusion

Epidemic perceived risk will increase anxiety in public, and the public who trust medical staff and the ability of the government to prevent and control the epidemic will have a higher PCSF. Compared with those in the outbreak stage of COVID-19, anxiety, PCSF, HSC and BF all decreased in the stable stage of COVID-19. HSC partly plays a mediating role in the process of the influence of PCSF on BF, which is not affected by the development of the epidemic. In the stable stage of COVID-19, the positive effect of PCSF on HSC is affected by anxiety. The moderated mediation model is verified. The epidemic situation is an important opportunity to enhance the PCSF in public. The common concept of a community with shared future for doctor-patient integrated into the values of life to shape, can be more solid, long-term maintenance of a good doctor-patient relationship. In addition, even if the epidemic is under control, mental health intervention should be maintained for a while or individualized intervention.

## Electronic supplementary material

Below is the link to the electronic supplementary material.

Supplementary Material 1

Supplementary Material 2

## Data Availability

The data presented in this study are available on request from the corresponding author. The data are not publicly available due to privacy restrictions.

## Abbreviations

BF: Benefit Finding
CI: Confidence Interval
COVID-19: Corona Virus Disease 2019
GAD: Generalized Anxiety Disorder
HSC: Health Self-Consciousness
PCSF: Perceived a Community with Shared Future for Doctor-Patient
