# Supplementary material for: Perceived a community with shared future for doctor-patient and benefit finding: a moderated mediation model
Source: BMC Psychol. 2023 May 30;11:174. doi: 10.1186/s40359-023-01175-6 (PMC10228459; doi:10.1186/s40359-023-01175-6)
Supplement: Supplementary file 1 — Supplementary Material 1 [file 40359_2023_1175_MOESM1_ESM.docx]

Table S1: The revised 7-item generalized anxiety disorder scale in the outbreak stage of COVID-19*

| The Revised 7-Item Generalized Anxiety Disorder Scale (GAD-7) | | | | |
| --- | --- | --- | --- | --- |
| How often have you been bothered by the following problems due to the current round of epidemic in your city? | None | A few days | More than a week | Almost every day |
| 1. Feel nervous, anxious or on edge | 1 | 2 | 3 | 4 |
| 2. Not being able to stop or control worrying | 1 | 2 | 3 | 4 |
| 3. Worrying too much about different things | 1 | 2 | 3 | 4 |
| 4. Trouble relaxing | 1 | 2 | 3 | 4 |
| 5.Being so restless that it is hard to sit still | 1 | 2 | 3 | 4 |
| 6.Becoming easily annoyed or irritable | 1 | 2 | 3 | 4 |
| 7. Feeling afraid as if something awful might happen | 1 | 2 | 3 | 4 |

* According to the GAD-8 classification standard, the option "None" to "Almost every day” is reassigned from 0 to 3 when the total score of the option is calculated.

Table S2: The revised 7-item generalized anxiety disorder scale in the stable stage of COVID-19*

| The Revised 7-Item Generalized Anxiety Disorder Scale (GAD-7) | | | | |
| --- | --- | --- | --- | --- |
| How often have you been bothered by the following problems due to the epidemic situation a while ago in your city? | None | A few days | More than a week | Almost every day |
| 1. Feel nervous, anxious or on edge | 0 | 1 | 2 | 3 |
| 2. Not being able to stop or control worrying | 0 | 1 | 2 | 3 |
| 3. Worrying too much about different things | 0 | 1 | 2 | 3 |
| 4. Trouble relaxing | 0 | 1 | 2 | 3 |
| 5.Being so restless that it is hard to sit still | 0 | 1 | 2 | 3 |
| 6.Becoming easily annoyed or irritable | 0 | 1 | 2 | 3 |
| 7. Feeling afraid as if something awful might happen | 0 | 1 | 2 | 3 |

* According to the GAD-8 classification standard, the option "None" to "Almost every day” is reassigned from 0 to 3 when the total score of the option is calculated.

Table S3: The revised benefit finding scale in the outbreak and the stable stages of COVID-19*

| The Revised Benefit Finding Scale(BFS) | | | | | |
| --- | --- | --- | --- | --- | --- |
|  | Fully agree | Moderately agree | Uncertain | Moderately disagree | Fully disagree |
| 1. Experiencing the epidemic has made me more aware of the significance of my learning to society. | 1 | 2 | 3 | 4 | 5 |
| 2. Experiencing the epidemic has made me realize that things are changeable and I have to do something more valuable in the future. | 1 | 2 | 3 | 4 | 5 |
| 3. Experiencing the epidemic has made me have a clearer plan for the rest of my life and study. | 1 | 2 | 3 | 4 | 5 |

*The scale was scored in reverse. When the scale is filled in online, the option “Fully disagree to “Fully agree” is scored 1 to 5. In order to facilitate the interpretation of the analysis results (the higher the scores, the higher benefit finding), the option “Fully disagree” to “Fully agree” will be reassigned from 1 to 5 when the total score of the options is calculated.

Table S4: The revised health self-consciousness scale in the outbreak and the stable stages of COVID-19*

| The Revised Health Self-Consciousness Scale (HSCS) | | | | | |
| --- | --- | --- | --- | --- | --- |
|  | Fully agree | Moderately agree | Uncertain | Moderately disagree | Fully disagree |
| 1. Affected by the epidemic, now I pay more attention to personal hygiene habits. | 1 | 2 | 3 | 4 | 5 |
| 2. Affected by the epidemic, now I pay more attention to a healthy lifestyle. | 1 | 2 | 3 | 4 | 5 |
| 3. Affected by the epidemic, now I’m more concerned about my health status. | 1 | 2 | 3 | 4 | 5 |

*The scale was scored in reverse. When the scale is filled in online, the option “Fully disagree to “Fully agree” is scored 1 to 5. In order to facilitate the interpretation of the analysis results (the higher the scores, the higher health self-consciousness), the option “Fully disagree” to “Fully agree” will be reassigned from 1 to 5 when the total score of the option is calculated.

Table S5: Perceived a community with shared future for doctor-patient scale during the epidemic and after the epidemic control*

| Perceived a Community with Shared Future for Doctor-Patient Scale (PCSFS) | | | | | |
| --- | --- | --- | --- | --- | --- |
|  | Fully agree | Moderately agree | Uncertain | Moderately disagree | Fully disagree |
| 1. Experiencing the epidemic has made me realize more deeply that the common enemy between doctors and patients is disease. | 1 | 2 | 3 | 4 | 5 |
| 2. Experiencing the epidemic has made me realize more deeply that coping with the disease requires the joint efforts of doctors and patients. | 1 | 2 | 3 | 4 | 5 |
| 3. Experiencing the epidemic has made me more aware of the limitations of modern medical technology. | 1 | 2 | 3 | 4 | 5 |

*The scale was scored in reverse. When the scale is filled in online, the option “Fully disagree to “Fully agree” is scored 1 to 5. In order to facilitate the interpretation of the analysis results (the higher the scores, the higher perceived a community with shared future for doctor-patient), the option from “Fully disagree” to “Fully agree” will be reassigned from 1 to 5 when the total score of the option is calculated.
