# Supplementary material for: Perceived a community with shared future for doctor-patient and benefit finding: a moderated mediation model
Source: BMC Psychol. 2023 May 30;11:174. doi: 10.1186/s40359-023-01175-6 (PMC10228459; doi:10.1186/s40359-023-01175-6)
Supplement: Supplementary file 2 — Supplementary Material 2 [file 40359_2023_1175_MOESM2_ESM.docx]

[Supplementary File 2](https://submission.springernature.com/submission/dd589a6b-75c3-41b7-a3c7-cff2c3023b62/file/55b9b456-ea24-4090-968a-99b01f92543f)：

The development time of the epidemic and the time of the two survives in different cities

|  | The time of the first confirmed case | Time of the first survey | Time of no new infections | Time of second survey |
| --- | --- | --- | --- | --- |
| Beijing | 2021/10/19 | 2021/11/14 | 2021/11/16 | — |
| Shangrao | 2021/10/30 | 2021/11/13 | 2021/11/15 | 2021/12/06 |
| Heihe | 2021/10/27 | 2021/11/13 | 2021/11/16 | 2021/12/07 |
| Dalian | 2021/11/04 | 2021/11/14 | 2021/11/28 | 2021/12/19 |
| Lanzhou | 2021/10/19 | — | 2021/11/10 | 2021/12/01 |
| Zhengzhou | 2021/11/03 | 2021/11/14 | 2021/11/19 | 2021/12/10 |
